# Supplementary material for: Structural basis of human transcription–DNA repair coupling
Source: Nature. 2021 Sep 15;598(7880):368–72. doi: 10.1038/s41586-021-03906-4 (PMC8514338; doi:10.1038/s41586-021-03906-4)
Supplement: Supplementary file 1 — The file contains Supplementary Fig. 1 and Supplementary Table 1. Supplementary Fig. 1 contains uncropped gel scans for Figs. 2a, b and 3a, and Extended Data Figs. 1a, b, f, 8a and 9a. Supplementary Table 1 contains source data associated with graphs for Extended Data Fig. 1a, b, f, h. [file 41586_2021_3906_MOESM1_ESM.pdf]

nature portfolio

https://doi.org/10.1038/s41586-021-03906-4

Supplementary information

Structural basis of human transcription–DNA repair coupling

In the format provided by the authors and unedited

Supplementary Figure 1 | Ucropped gel images

Figure 2a

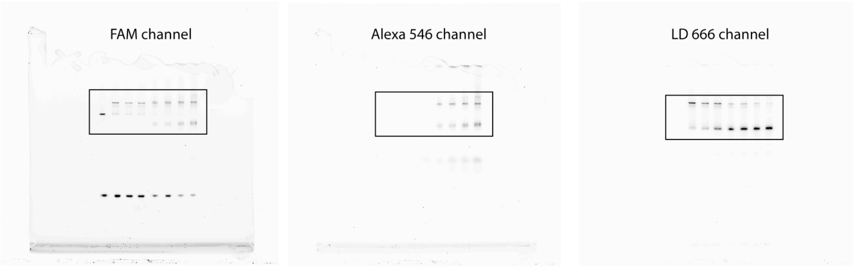

Figure 2b

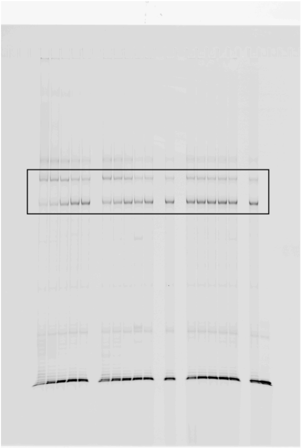

Extended Data Figure 1a

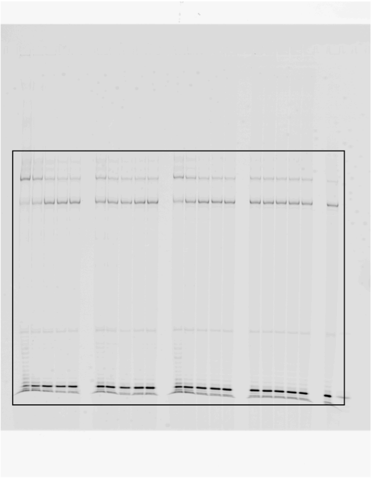

Extended Data Figure 1b (right) and 1f (left)

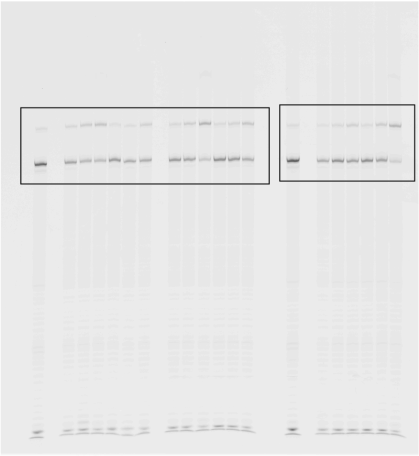

Figure 3a

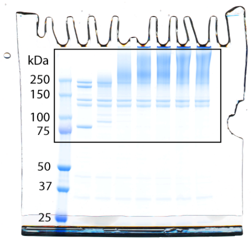

Extended Data Figure 8a

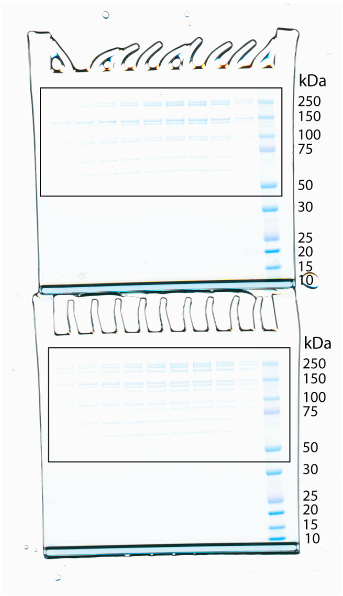

Extended Data Figure 9a

Western blot signal

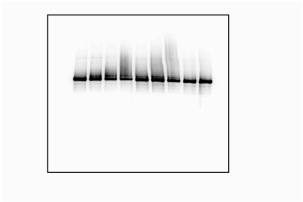

Marker

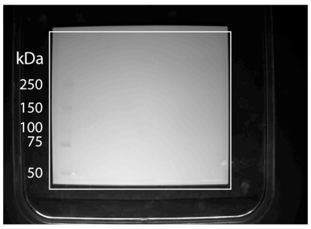

Supplementary Table 1 | Supplementary Source Data

Extended Data Figure 1a

Gel 1:	Arrest	Bypass	Background	Arrest-Background	Bypass-Background	Bypass / Arrest
	16598,20	13475,11	13017,00	3581,20	458,11	0,13
	18847,40	14176,13	13017,00	5830,40	1159,13	0,20
	20463,09	14364,52	13017,00	7446,09	1347,52	0,18
	20703,62	14370,35	13017,00	7686,62	1353,35	0,18
	21519,55	14638,65	13017,00	8502,55	1621,65	0,19
	20883,29	14567,85	13017,00	7866,29	1550,85	0,20
	19581,79	14363,41	13017,00	6564,79	1346,41	0,21
	19508,71	14441,36	13017,00	6491,71	1424,36	0,22
	18713,85	15357,71	13017,00	5696,85	2340,71	0,41
	16678,22	16265,05	13017,00	3661,22	3248,05	0,89
	16409,26	16251,79	13017,00	3392,26	3234,79	0,95
	19226,62	14511,65	13017,00	6209,62	1494,65	0,24
	19255,56	14688,77	13017,00	6238,56	1671,77	0,27
	19034,01	15071,42	13017,00	6017,01	2054,42	0,34
	17976,29	15577,36	13017,00	4959,29	2560,36	0,52
	17281,89	16121,45	13017,00	4264,89	3104,45	0,73
	20877,19	15059,31	13017,00	7860,19	2042,31	0,26
	20966,19	15203,08	13017,00	7949,19	2186,08	0,28
	18191,32	16354,85	13017,00	5174,32	3337,85	0,65
	15742,80	17946,37	13017,00	2725,80	4929,37	1,81
	16399,44	18662,71	13017,00	3382,44	5645,71	1,67

Gel 2:	Arrest	Bypass	Background	Arrest-Background	Bypass-Background	Bypass / Arrest
	15077,52	12772,97	12519,60	2557,92	253,37	0,10
	17754,71	13312,09	12519,60	5235,11	792,49	0,15
	17721,40	13393,97	12519,60	5201,80	874,37	0,17
	18287,04	13457,27	12519,60	5767,44	937,67	0,16
	17212,79	13387,79	12519,60	4693,19	868,19	0,18
	20692,83	14129,04	12519,60	8173,23	1609,44	0,20
	17863,29	13563,76	12519,60	5343,69	1044,16	0,20
	18014,53	13639,52	12519,60	5494,93	1119,92	0,20
	17200,60	13994,54	12519,60	4681,00	1474,94	0,32
	16254,19	14934,39	12519,60	3734,59	2414,79	0,65
	15751,12	15091,77	12519,60	3231,52	2572,17	0,80
	18658,64	13900,15	12519,60	6139,04	1380,55	0,22
	18546,55	14094,75	12519,60	6026,95	1575,15	0,26
	18040,13	14206,65	12519,60	5520,53	1687,05	0,31
	19990,08	15666,74	12519,60	7470,48	3147,14	0,42

	17558,32	14844,60	12519,60	5038,72	2325,00	0,46
	18284,04	13899,25	12519,60	5764,44	1379,65	0,24
	19209,56	14151,90	12519,60	6689,96	1632,30	0,24
	17259,64	14743,71	12519,60	4740,04	2224,11	0,47
	14890,62	16101,19	12519,60	2371,02	3581,59	1,51
	16454,53	18505,91	12519,60	3934,93	5986,31	1,52

Gel 3:	Arrest	Bypass	Background	Arrest-Background	Bypass-Background	Bypass / Arrest
	14207,48	10697,32	10350,23	3857,26	347,09	0,09
	14268,11	10986,20	10350,23	3917,89	635,98	0,16
	16111,87	11237,07	10350,23	5761,65	886,84	0,15
	15467,33	11148,70	10350,23	5117,11	798,48	0,16
	15389,34	11154,91	10350,23	5039,12	804,68	0,16
	12264,56	10571,59	10350,23	1914,34	221,37	0,12
	15018,03	11038,18	10350,23	4667,81	687,96	0,15
	14516,15	10987,08	10350,23	4165,93	636,86	0,15
	14797,46	11818,42	10350,23	4447,24	1468,20	0,33
	12255,15	11898,33	10350,23	1904,93	1548,11	0,81
	12903,09	12420,85	10350,23	2552,87	2070,63	0,81
	14369,54	10821,69	10350,23	4019,32	471,47	0,12
	14310,38	10923,74	10350,23	3960,16	573,51	0,14
	14371,50	11248,08	10350,23	4021,28	897,86	0,22
	13193,05	11386,84	10350,23	2842,83	1036,62	0,36
	12232,09	11284,15	10350,23	1881,87	933,92	0,50
	14894,75	10903,23	10350,23	4544,53	553,00	0,12
	13474,88	10750,14	10350,23	3124,66	399,91	0,13
	12830,22	11677,89	10350,23	2480,00	1327,67	0,54
	11747,35	12829,48	10350,23	1397,13	2479,26	1,77
	11243,67	11895,51	10350,23	893,45	1545,29	1,73

Extended Data Figure 1b

Gel 1:	Arrested	Bypass	Background	Arrested-Background	Bypass-Background	Bypass/Arrest
	10845,08	4143,594	3192,828	7652,252	950,766	0,124246562
	6861,507	3992,49	3192,828	3668,679	799,662	0,21797001
	8242,056	4171,756	3192,828	5049,228	978,928	0,193876767
	7192,465	4630,257	3192,828	3999,637	1437,429	0,359389865
	8190,376	4179,051	3192,828	4997,548	986,223	0,197341376
	6899,572	4584,691	3192,828	3706,744	1391,863	0,375494774
	4943,623	5861,065	3192,828	1750,795	2668,237	1,524014519

Gel 2:	Bypass	Arrested	Background	Bypass-Background	Arrested-Background	Bypass/Arrest
	7007,592	17491,618	5551,051	1456,541	11940,567	0,121982566
	6754,002	10801,072	5551,051	1202,951	5250,021	0,229132607
	7100,345	13014,182	5551,051	1549,294	7463,131	0,207593033
	7728,522	11756,678	5551,051	2177,471	6205,627	0,350886542
	7018,609	13128,217	5551,051	1467,558	7577,166	0,193681648
	7964,267	11713,918	5551,051	2413,216	6162,867	0,391573597
	9834,619	8466,195	5551,051	4283,568	2915,144	1,469419006

Extended Data Figure 1f

Gel 1:	Arrested	Bypass	Background	Arrested-Background	Bypass-Background	Bypass/Arrest
	11014,099	4279,178	3192,828	7821,271	1086,35	0,138896862
	7401,125	4292,115	3192,828	4208,297	1099,287	0,261218968
	5969,6	4728,349	3192,828	2776,772	1535,521	0,552987786
	6013,226	5289,181	3192,828	2820,398	2096,353	0,743282686
	8001,517	4334,208	3192,828	4808,689	1141,38	0,237357833
	7351,52	4166,117	3192,828	4158,692	973,289	0,234037289
	7338,581	4730,138	3192,828	4145,753	1537,31	0,370815627
	7952,121	4273,792	3192,828	4759,293	1080,964	0,227127012
	7385,704	4782,054	3192,828	4192,876	1589,226	0,37903005
	5459,253	6073,669	3192,828	2266,425	2880,841	1,271094786
	7970,171	4083,306	3192,828	4777,343	890,478	0,186396078
	8417,011	4588,288	3192,828	5224,183	1395,46	0,267115451
	6936,594	5211,072	3192,828	3743,766	2018,244	0,539094591

Gel 2:	Bypass	Arrested	Background	Bypass-Background	Arrest-Background	Bypass/Arrest
	7032,744	16207,859	5551,051	1481,693	10656,808	0,139037224
	7105,702	11537,269	5551,051	1554,651	5986,218	0,259705042
	7967,56	9933,522	5551,051	2416,509	4382,471	0,551403306
	8716,105	9578,896	5551,051	3165,054	4027,845	0,785793396
	6828,592	12163,638	5551,051	1277,541	6612,587	0,193198365
	6692,575	10482,717	5551,051	1141,524	4931,666	0,23146823
	7597,677	10792,836	5551,051	2046,626	5241,785	0,390444476
	7161,159	12355,074	5551,051	1610,108	6804,023	0,236640587
	7883,958	11269,946	5551,051	2332,907	5718,895	0,407929679
	9921,152	8846,943	5551,051	4370,101	3295,892	1,325923604
	7170,359	13556,043	5551,051	1619,308	8004,992	0,202287273
	7775,074	12657,583	5551,051	2224,023	7106,532	0,312954758
	8505,375	10627,705	5551,051	2954,324	5076,654	0,581943146

Extended Data Figure 1h

ATPase rate for replicates is reported (nM/min)

Sample	Replicate 1	Replicate 2	Replicate 3
CsB	7,9	5,0	9,5
CsB+DNA	30,0	24,0	25,9
CsB+EC	29,6	23,2	31,9
CsB F796A	6,2	3,2	7,3
CsB F796A+DNA	22,1	17,7	20,0
CsB F796A+EC	18,7	17,0	18,3
CsB+CsA+DDB1+UVSSA+EC	43,2	37,9	47,1
CsB+CsA+DDB1+UVSSA+EC+PAF	46,4	43,4	53,5
