# Supplementary material for: Structural basis of human transcription–DNA repair coupling
Source: Nature. 2021 Sep 15;598(7880):368–72. doi: 10.1038/s41586-021-03906-4 (PMC8514338; doi:10.1038/s41586-021-03906-4)
Supplement: Supplementary file 2 — Reporting Summary [file 41586_2021_3906_MOESM2_ESM.pdf]

nature research

Corresponding author(s): Cramer, Patrick

Last updated by author(s): Jun 18, 2021

Reporting Summary

Nature Research wishes to improve the reproducibility of the work that we publish. This form provides structure for consistency and transparency in reporting. For further information on Nature Research policies, see our Editorial Policies and the Editorial Policy Checklist.

Statistics

For all statistical analyses, confirm that the following items are present in the figure legend, table legend, main text, or Methods section.

n/a	Confirmed	
☐	☒	The exact sample size (*n*) for each experimental group/condition, given as a discrete number and unit of measurement
☒	☐	A statement on whether measurements were taken from distinct samples or whether the same sample was measured repeatedly
☒	☐	The statistical test(s) used AND whether they are one- or two-sided<br>*Only common tests should be described solely by name; describe more complex techniques in the Methods section.*
☒	☐	A description of all covariates tested
☒	☐	A description of any assumptions or corrections, such as tests of normality and adjustment for multiple comparisons
☐	☒	A full description of the statistical parameters including central tendency (e.g. means) or other basic estimates (e.g. regression coefficient) AND variation (e.g. standard deviation) or associated estimates of uncertainty (e.g. confidence intervals)
☒	☐	For null hypothesis testing, the test statistic (e.g. *F*, *t*, *r*) with confidence intervals, effect sizes, degrees of freedom and *P* value noted<br>*Give P values as exact values whenever suitable.*
☒	☐	For Bayesian analysis, information on the choice of priors and Markov chain Monte Carlo settings
☒	☐	For hierarchical and complex designs, identification of the appropriate level for tests and full reporting of outcomes
☒	☐	Estimates of effect sizes (e.g. Cohen's *d*, Pearson's *r*), indicating how they were calculated

Our web collection on statistics for biologists contains articles on many of the points above.

Software and code

Policy information about availability of computer code

Data collection	Serial EM 3.8 beta 8
Data analysis	RELION 3.0 beta-2, UCSF Chimera 1.13, UCSF ChimeraX v0.8, Coot 0.9, Warp v1.0.7, PHENIX 1.18, cryoSPARC 2.14.2, Prism 9, ImageJ version 1.47v, Molprobity 4.5.1, XlinkAnalyzer version 1.1

For manuscripts utilizing custom algorithms or software that are central to the research but not yet described in published literature, software must be made available to editors and reviewers. We strongly encourage code deposition in a community repository (e.g. GitHub). See the Nature Research guidelines for submitting code & software for further information.

Data

Policy information about availability of data

All manuscripts must include a data availability statement. This statement should provide the following information, where applicable:

- Accession codes, unique identifiers, or web links for publicly available datasets
- A list of figures that have associated raw data
- A description of any restrictions on data availability

The electron density reconstructions and structure coordinates were deposited with the Electron Microscopy Database (EMDB) and with the Protein Data Bank (PDB) under the following accession codes: PDB code 7OO3 and EMDB-13004 for Structure 1, PDB code 7OOB and EMDB-13009 for Structure 2, PDB code 7OOP and EMDB-13010 for Structure 3, PDB code 7OPC and EMDB-13015 for Structure 4 and PBD code 7OPD and EMDB-13016 for Structure 5. The crosslinking mass spectrometric data and the ubiquitin-mapping data have been deposited to the ProteomeXchange Consortium via the PRIDE with the dataset identifier PXD025328 .

Field-specific reporting

Please select the one below that is the best fit for your research. If you are not sure, read the appropriate sections before making your selection.

☒ Life sciences ☐ Behavioural & social sciences ☐ Ecological, evolutionary & environmental sciences

For a reference copy of the document with all sections, see nature.com/documents/nr-reporting-summary-flat.pdf

Life sciences study design

All studies must disclose on these points even when the disclosure is negative.

Sample size	No statistical methods were used to predetermine sample size. All biochemical experiments were replicated two or more times. Structural data was collected on five independently prepared samples.
Data exclusions	No data were excluded from the analyses.
Replication	All attempts at replication were successful, at least two repetitions for biochemical assays were performed. Cryo-EM single particle analysis inherently relies on averaging over a large number of independent observations.
Randomization	Samples were not allocated to groups.
Blinding	Investigators were not blinded during data acquisition and analysis because it is not a common procedure for the methods employed.

Reporting for specific materials, systems and methods

We require information from authors about some types of materials, experimental systems and methods used in many studies. Here, indicate whether each material, system or method listed is relevant to your study. If you are not sure if a list item applies to your research, read the appropriate section before selecting a response.

Materials & experimental systems

n/a	Involved in the study	
☐	☒	Antibodies
☐	☒	Eukaryotic cell lines
☒	☐	Palaeontology and archaeology
☒	☐	Animals and other organisms
☒	☐	Human research participants
☒	☐	Clinical data
☒	☐	Dual use research of concern

Methods

n/a	Involved in the study	
☒	☐	ChIP-seq
☒	☐	Flow cytometry
☒	☐	MRI-based neuroimaging

Antibodies

Antibodies used	F-12 Pol II antibody, Santa Cruz Biotechnology, sc-55492; anti-mouse HRP conjugate, Abcam, ab5870
Validation	Mouse monoclonal antibody to RNA polymerase II subunit A. Antibody has been validated for the following applications: EIA, Immunoassay, Precipitation, ELISA, Immunofluorescence, Immunohistochemistry - fixed, Immunoprecipitation, and Western Blot. Here it was used for Western blot.<br><br>Anti-mouse HRP conjugate was used as a secondary antibody.

Eukaryotic cell lines

Policy information about cell lines

Cell line source(s)	Hi5 cells: Expression Systems, Tni Insect cells in ESF921 media, item 94-002F<br>Sf9 cells: ThermoFisher, Catalogue Number 12659017, Sf9 cells in Sf-9000TM III SFM<br>Sf21 cells: Expression Systems, SF21 insect cells in ESF921 medium, Item 94-003F
Authentication	None of the cell lines were authenticated.
Mycoplasma contamination	Cell lines were not tested for mycoplasma contamination.
Commonly misidentified lines (See ICLAC register)	No commonly misidentified cell lines were used.
